# Effects of MICT and HIIT on mitochondrial dynamics-related proteins in visceral adipose tissue of type 2 diabetic rats

**DOI:** 10.1186/s40659-026-00694-x

**Published:** 2026-04-24

**Authors:** Mohammad Rahman Rahimi, Ian G. Davies, Hadi Golpasandi, Narges Fatahi

**Affiliations:** 1 Department of Exercise Physiology, University of Kurdistan, Sanandaj, 66177- 15175 Iran; 2 Research Institute of Sport and Exercise Science, Liverpool John Moores University, Liverpool, UK

**Keywords:** Mitochondrial dynamics, MICT, HIIT, Insulin resistance, Type 2 diabetes

## Abstract

**Background:**

Adipose tissue, as an endocrine gland, significantly influences the pathophysiology of type 2 diabetes (T2DM) through mitochondrial dysfunction. This study examined the effects of two exercise training types on mitochondrial dynamic protein content in visceral adipose tissue (VAT) of T2DM-induced rats.

**Methods:**

Male Wistar rats (total *n* = 40; 8–10 weeks old, ~ 220 g) were used. Thirty-two rats were fed a high-fat diet (HFD) for 6 weeks and received streptozotocin (STZ) to induce type 2 diabetes mellitus (T2DM). Eight additional rats were maintained on standard chow as the healthy control group (NC, *n* = 8). The diabetic rats were randomly divided into three groups (*n* = 8 each): diabetic control (DC), diabetes + MICT (MICT + D), and diabetes + HIIT (HIIT + D). All animals (total *n* = 40) completed the study and were included in the analyses. MICT involved 8 weeks of training (5 sessions/week, 50 min at 60% maximal speed), while HIIT consisted of 8 weeks (5 sessions/week, 4–7 sets of 4 min at 80–90% maximal speed). Western blot analysis revealed significant increases in mitochondrial fission proteins (Drp-1: 238%, Fis-1: 99%) in the DC group compared to HC (*p* < 0.05).

**Results:**

Both MICT and HIIT significantly reduced Drp-1 (53.55%, 77.81%) and Fis-1 (16.08%, 36.18%) while increasing MFN-2 (26.25%, 57.50%) and OPA-1 (66.10%, 103.39%) compared to DC. HIIT showed greater changes than MICT (*p* < 0.05). Additionally, fasting serum glucose and HOMA-IR levels were significantly lower in both exercise groups (*p* < 0.001).

**Conclusions:**

In conclusion, Type 2 diabetes causes changes in the levels of proteins regulating mitochondrial fission and fusion in visceral adipose tissue, which can be improved through MICT and HIIT training, potentially increasing aerobic capacity and glucose metabolism.

**Supplementary Information:**

The online version contains supplementary material available at 10.1186/s40659-026-00694-x.

## Background

Type 2 diabetes mellitus (T2DM) is a chronic metabolic disease characterized by insulin resistance, elevated blood glucose levels, and impaired lipid metabolism [1]. The disease is directly linked to a malfunction in mitochondria, a structure that plays a key role in maintaining metabolic health and energy regulation [2]. Adipose tissue (AT), as an active endocrine organ, is instrumental in the pathophysiology of T2DM, as elevated, especially visceral AT, is linked to metabolic disruption including dyslipidaemia, poor glucose uptake, and altered adipokine secretion [3, 4]. AT mitochondria are of particular importance in the development of diabetes and its associated complications, as they regulate energy expenditure and oxidative stress [5].

Maintaining the function and health of mitochondria involves the processes of fission and fusion dynamics [6]. Proteins such as Fission-1 (FIS1) and Dynamin-related protein-1 (DRP1) facilitate the process of mitochondrial fission, which enables the separation of damaged mitochondria and helps in mitophagy. Regarding mitochondrial fusion, the proteins Optic Atrophy 1 (OPA1) and Mitofusin-2 (MFN2) help maintain the optimal function of mitochondria. Disruption in the regulation of the expression of these proteins can cause an imbalance in the processes of fission and fusion, and consequently lead to mitochondrial dysfunction in T2DM [7]. Exercise has been considered one of the most effective non-pharmacological approaches for diabetes control or possible remission [8]. Exercise improves insulin sensitivity, promoting glucose metabolism, and enhancing mitochondrial function [9]. Among the different types of exercise training, moderate-intensity continuous training (MICT) and high-intensity interval training (HIIT) have attracted particular attention due to their distinct and significant effects on metabolic and physiological adaptations [10]. MICT involves sustained, continuous moderate-intensity exercise that increases mitochondrial biogenesis and improves muscle oxidative capacity [11]. In contrast, HIIT involves alternating short bursts of high-intensity activity with periods of low-intensity recovery. This type of training stimulates mitochondrial repair and improves metabolic flexibility by generating physiological stress signals [12]. Both types of exercise help combat metabolic disorders associated with diabetes in different ways and are effective in promoting the metabolic health of people living with T2DM [13].

Despite substantial evidence supporting the metabolic benefits of MICT and HIIT, the differential effects of these two protocols on regulatory proteins of mitochondrial fission and fusion in adipose tissue; particularly visceral adipose tissue (VAT); in the context of type 2 diabetes remain unknown [14–16].

Recently, a six week study of HIIT and MICT training showed both modalities significantly increased the expression of genes involved in mitochondrial dynamics, such as *MFN2* mRNA, *OPA1* mRNA, *DRP1* mRNA, and *FIS1* mRNA, in the soleus skeletal muscle and abductor digitorum longus in rats, with no difference between the two types of training protocols [17]. In another study, induced obesity reduced *MFN2* and *DRP1* levels in soleus muscle, while both HIIT and MICT increased them - with HIIT showing significantly greater effects than MICT [18]. However, findings from studies on the differential effects of HIIT and MICT on changes in the levels of proteins regulating mitochondrial fission and fusion are inconsistent. While some studies report distinct adaptations—such as HIIT causing greater upregulation of fusion proteins (MFN2, OPA1) and downregulation of fission markers (DRP1, FIS1) compared with MICT [19], others observed similar improvements in both protocols without significant differences. These discrepancies may be due to variations in experimental designs (e.g., exercise duration, intensity protocols, or participant characteristics) and highlight the need for further research to elucidate the specific mechanisms underlying mitochondrial remodeling [20]. Therefore, studying the regulation of mitochondrial fission and fusion processes and evaluating key proteins such as FIS1, DRP1, OPA1, and MFN2 may clarify the molecular mechanisms associated with metabolic adaptations in response to exercise modality. Deeper insights of these mechanisms can provide a suitable platform for designing targeted exercise interventions, leading to improved mitochondrial function and reduced complications associated with T2DM, advance the existing knowledge in the field of mitochondrial biology, and provide practical and effective solutions for controlling T2DM and improving the quality of life of people living with this disease.

The present study addresses this gap by examining the effects of MICT and HIIT on the expression of key proteins regulating mitochondrial dynamics (FIS1, DRP1, OPA1, and MFN2) in visceral adipose tissue of streptozotocin/high-fat diet-induced type 2 diabetic rats. This work is novel because it focuses on VAT, a critical but understudied metabolic tissue in mitochondrial dynamics research, rather than skeletal muscle, which has dominated the scientific literature. By elucidating potential tissue-specific adaptations, our findings may reveal distinct mechanisms through which exercise regimens improve metabolic outcomes in T2DM and provide a basis for designing more targeted, tissue-specific exercise interventions.

## Methods

### Study design and ethical approval

The research design was a randomized controlled trial (RCT) with four groups of rats including a healthy normal control group (HC), diabetic control group (DC), diabetes + moderate-intensity continuous exercise (D + MICT) group, and diabetes +high-intensity interval training (D + HIIT) group. Ethical approvals for the use and care of animals were obtained from the Ethics Committee of the University of Kurdistan (IR.UOK.REC.1404.018), following the Arrive Standard Guidelines for the Humane Treatment of Laboratory Animals.

## Rat selection and maintenance guidelines

Male Wistar rats (RRID: RGD_737960) (*n* = 36), 8 to 10 weeks old and weighing 220 to 250 g, were purchased from the Pasteur Institute of Karaj. The sample size was determined using G*Power software (version 3.1.9, Germany) with an effect size of 0.62, an alpha level of 0.05, and a statistical power of 0.80. The effect size selected was based on Cohen’s conventional effect size criteria, which indicates a medium to large expected effect for behavioral and physiological outcomes in exercise-based interventions [21]. The primary outcome was the change in protein expression levels of FIS1, MFN2, OPA1, and DRP-1 to provide a comprehensive picture of changes in fission and fusion regulatory proteins. The effect size (Cohen’s d = 0.62) was selected based on Cohen’s conventional criteria, which indicates a medium to large effect (Cohen, 1988). This value was considered appropriate for detecting biologically significant changes in the expression of proteins related to mitochondrial dynamics in animal models of type 2 diabetes and exercise interventions, as such models typically show medium to large differences in these proteins. The calculated sample size for each group was *n* = 8.

**Fig. 1:**
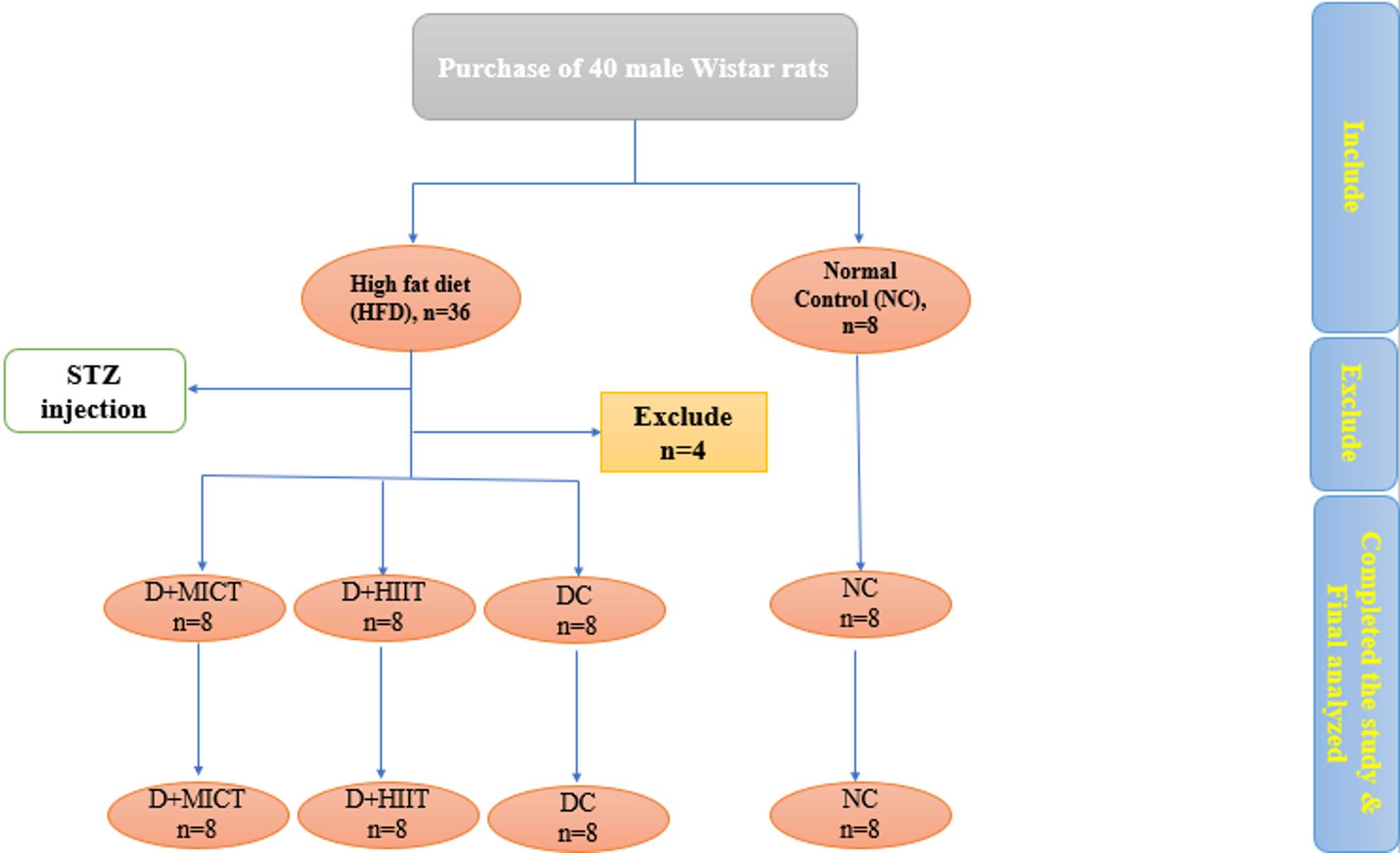
Flow diagram of study participants (rats) from acquisition to data analysis

Rats were maintained in a temperature-controlled environment (22–24 °C) with a 12-hour light-dark cycle. Standard chow and water were provided ad libitum throughout the experimental period. Rats were randomly assigned to a standard diet (*n* = 8) or a high-fat diet (*n* = 28), (Fig. 1).

## T2DM induction model

Rats in the diabetic groups (DC, D+MICT, D+HIIT) were fed a standardized high-fat diet (HFD) for 4 weeks prior to diabetes induction. The HFD consisted of 45% fat, 35% carbohydrate, and 20% protein (energy density ≈ 5.2 kcal/g or 21.8 kJ/g). Food was provided ad libitum, and daily caloric intake was monitored and adjusted to ensure consistent weight gain across the diabetic groups. After 4 weeks of HFD feeding, these rats received a single intraperitoneal injection of low-dose streptozotocin (STZ; 30 mg/kg body weight) dissolved in citrate buffer (pH 4.4) to induce type 2 diabetes mellitus.

The healthy control group (HC) was maintained on a standard chow diet (≈ 3.0 kcal/g) throughout the entire study period and received an equivalent volume of vehicle (citrate buffer without STZ) at the same time point as the diabetic groups to control for procedural and stress-related effects.

Following diabetes induction, all diabetic groups continued their respective diets (HFD for diabetic groups and standard chow for HC) until the end of the 8-week intervention period. Daily food intake was monitored, and any unconsumed food was collected and weighed to ensure accurate recording of energy and macronutrient consumption (Fig. 1) [22].

## Streptozotocin (STZ) injection

After four weeks of high-fat diet (HFD) feeding, rats in the diabetic groups (DC, MICT + D, HIIT + D) received an intraperitoneal injection of a low dose of streptozotocin (STZ; 30 mg/kg body weight) dissolved in citrate buffer (pH 4.4) to induce type 2 diabetes mellitus (T2DM). The healthy control group (HC) received an equivalent volume of vehicle (citrate buffer, pH 4.4) with the same injection and handling method to control for stress-related and procedural confounders. One week after injection, fasting blood glucose levels were measured using a glucometer (Accu-Chek Performa; Roche Diagnostics, USA). Rats with fasting blood glucose > 16.7 mmol/L were diagnosed as diabetic and randomly selected. During the diabetes induction phase (STZ injection), four rats died due to complications related to streptozotocin administration (hypoglycemia or acute toxicity) and were excluded from the study before randomization. No animals were excluded after randomization or during the 8-week intervention period. The final sample size analyzed was *n* = 8 per group (total *n* = 32 diabetic rats + *n* = 8 healthy rats). (Fig. 1). All procedures were performed in accordance with the ethical principles for the care and use of laboratory animals and approved by the Research Ethics Committee of the University of Kurdistan under the number IR.UOK.REC.1404.018.

## Grouping of rats

After induction of T2DM, rats were randomly divided into 4 groups: (1) DC (*n* = 8), (2) D+HIIT (*n* = 8), and 4) D+MICT (*n* = 8). Randomization of rats was performed to ensure non-biased allocation to groups and to reduce selection bias. Each rat was individually marked with a tag on its back for unique identification. 32 diabetic rats were randomly assigned to three diabetic groups (DC, D + MICT, D + HIIT) using an online randomization tool (https://randomizer.org/). Allocation was stratified by body weight to minimize baseline differences between groups. The randomization sequence was generated and assigned by an individual who was not involved in the outcome assessment to ensure allocation concealment. Cage effects were minimized by balancing the distribution of rats across different holding rack positions (random rotation of cages and balanced placement of treatment groups on different rack floors). The healthy control (HC) group was housed separately and did not participate in the randomization process with the diabetic groups. Rats in the training groups ran on a treadmill at five times a week for eight weeks as shown in detail below and Table 1. Rats in the HC and DC groups did not perform any exercise during the eight weeks.

A total of 40 male Wistar rats were used. Eight rats were fed a standard chow as healthy controls (HC). Thirty-two rats were fed a high-fat diet for 4 weeks, followed by streptozotocin injection to induce type 2 diabetes. Four rats died due to STZ-related complications before randomization and were excluded from the study. The remaining 32 diabetic rats were randomly divided into three groups (*n* = 8 each): diabetic control (DC), diabetes + MICT (MICT + D), and diabetes + HIIT (HIIT + D). No further dropouts or losses occurred during the intervention. All 40 rats were included in the final analysis (*n* = 8 per group). The animal allocation and exclusion flow is summarized in Fig. 1.

### Incremental exercise test (GXT)

Exercise capacity was assessed using an incremental treadmill test performed twice during the 8-week intervention (pretest at baseline and posttest at the end). The test consisted of 10 steps, each lasting 3 min, starting at a speed of 0.3 km/h with a 0% incline. The speed was increased by 0.3 km/h at the beginning of each subsequent step until the rat reached fatigue (defined as the inability to continue running despite gentle stimulation for more than 10 s). The maximum speed achieved before fatigue was recorded as the time to exhaustion (TTE) and used to determine maximal running velocity (Vmax). Training intensity for both MICT and HIIT groups was adjusted every 2 weeks based on the last Vmax measurement [23].

## Moderate intensity continuous training (MICT) protocol

Before beginning the MICT and HIIT exercise programs, all rats underwent a one-week treadmill acclimation, walking 10 min daily at 10 m per minute. The main training protocol consisted of eight weeks of aerobic training, five times a week, with 50–60% of V_max_ and an average of 50 min per session. Before the main protocol was implemented, a 5-minute warm-up by running on the treadmill was performed in each session. The changes in intensity and duration of training were based on the principle of overload, so that in the first week, the rats were exercised for 30 min at a speed of 50% of V_max_, then in the second week, the training duration increased to 35 min at an intensity of 52% of V_max_. In the third and fourth weeks, the intensity increased to 54% of V_max_ and the duration to 40 min. In weeks five and six, the duration increased to 45 and 50 min and the intensity to 56 and 58% of V_max_ respectively. In weeks seven and eight, the duration (50 min) and intensity (60% of V_max_) remained constant [24], (Table 1).

## High-intensity interval training (HIIT) protocol

The main body of the HIIT training protocol will consist of 4 min of running at 85–90% of your maximum speed and 3 min of running at 50–60% of your maximum speed, repeated seven times (totaling 49 min of training). A 5-minute warm-up and cool-down at 40% of your maximum speed will be performed before and after the training session [24]. It should be noted that both HIIT and CMIT training protocols were matched in terms of training volume (training time per session) (Table 1).

**Table 1:** HIIT and MICT volume and intensity

Num. weeks	HIIT		MICT
	High-intensity bout (num/min/intensity)	Rest interval bout (num/min/intensity)	Duration, Intensity (min/ %_max speed_)
1st week	4/4/85% _max speed_	4/3/50% _max speed_	30 min, 50% _max speed_
2nd week	5/4/ 85% _max speed_	5/3/50% _max speed_	35 min, 52% _max speed_
3rd week	6/4/85% _max speed_	6/3/53% _max speed_	40 min, 54% _max speed_
4th week	6/4/ 87% _max speed_	6/3/53% _max speed_	40 min, 54% _max speed_
5th week	7/4/ 87% _max speed_	7/3/56% _max speed_	45 min, 56% _max speed_
6th week	7/4/87% _max speed_	7/3/56% _max speed_	50 min, 58% _max speed_
7th week	7/4/90% _max speed_	7/3/60% _max speed_	50 min, 60% _max speed_
8th week	7/4/90% _max speed_	7/3/60% _max speed_	50 min, 60% _max speed_

### Anesthesia and tissue collection process

#### Euthanasia and tissue collection

At the end of the training period, rats were anesthetized after a fast (8 h) with a combination of code (301ED874F12) made in Belgium at a rate of 75 mg/kg and xylazine 10 mg/kg. After an 8-hour fast, rats were anaesthetised with ketamine (75 mg/kg) and xylazine (10 mg/kg), and then euthanised by an overdose of sodium pentobarbital (≥ 150 mg/kg, intraperitoneally) under deep anaesthesia, in accordance with institutional and national ethical guidelines (protocol number: IR.UOK.REC.1404.018). This method is consistent with the recommendations of the European Directive 2010/63/EU and ASPA Schedule 1 for humane killing of laboratory animals. Visceral adipose tissue was immediately removed, frozen in liquid nitrogen, and stored at -80 °C for further analysis.

### Protein isolation and immunoblotting assessment

To homogenize adipose tissue, the adipose tissue was first homogenized in lysis buffer containing protease and phosphatase inhibitors. Then, the Bradford method was used to determine the protein quantity and total protein concentration. For Western blot analysis, equal amounts of protein (30–50 µg) were first separated by SDS-PAGE and transferred onto PVDF membranes. The membranes were blocked with 5% nonfat dry milk in TBST and incubated at 4 °C with primary antibodies against (Drp-1 (C-5):sc-271583, RRID: AB_10659110, FIS1 (B-5):sc-376447, RRID: AB_11149382, sc-393296, RRID: AB_3101815, Opa1(D-9): and Mfn2 (Y-19) sc:30366, RRID: AB_2126309, B-actin (2A3):sc517582, RRID: AB_2833259, all from Santa Cruz Biotechnology. After washing, the membranes were incubated with HRP-conjugated secondary antibodies for 1 h at room temperature. Protein bands were visualized using enhanced chemiluminescence (ECL) and quantified with densitometric software [25].

Fasting blood glucose (FBG) levels were measured using a Mindrey BS200 colorimetric method and insulin levels were measured using an ELISA kit (Alpco, catalog number 80-INSRTH-E01, E10). HOMA-IR index was also calculated using the following formula [26].

HOMA-IR= Fasting Insulin (mmol/L) * Fasting Glucose (mmol/L) /22.5.

### Statistical method

Data were expressed as mean ± standard deviation (SD). The Shapiro-Wilk test was used to examine the normality of data distribution. Group comparisons of variables were examined using one-way analysis of variance, and Tukey’s post hoc test was used for multiple comparisons. The level of statistical significance was considered at the *p* < 0.05 level.

## Results

The mean weight, serum glucose, insulin levels, and HOMA-IR index are presented in Table 2.

**Table 2:** Mean ± SD of weight, serum glucose, insulin levels, and HOMA-IR index

Groups variables	HC		DC	D+MICT	D+HIIT
Weight (g)	382.4 ± 14.15		373.4 ± 12.70	^*^ 320.4 ± 11.66	^* †^ 278.5 ± 12.54
FBS (mmol/l)	4.42 ± 1.59		21.23 ± 5.81 ^¥^	15.23 ± 3.13^*^	13.03 ± 1.03^* †^
Insulin (mmol/l)	3.05 ± 1.23		6.75 ± 2.56^¥^	4.47 ± 2.10 ^∗^	3.65 ± 1.88 ^*^
Homa-IR	0.59 ± 0.18		6.36 ± 2.13 ^¥^	3.02 ± 1.84 ^∗^	2.11 ± 1.09 ^∗^
TTE (min)	Pre-Test	20.90 ± 1.93	20.15 ± 1.87	20.79 ± 2.20	20.87 ± 2.38
	Post-Test	18.58 ± 1.73	11.13 ± 1.94^¥^	29.42 ± 2.33^¥ *^	33.19 ± 2.56 ^¥ *^

*P* < 0.05.

(¥): Significant difference compared to HC group, (*): Significance vs. DC group, (†): Significant difference compared to DC group D+MICT (*P* < 0.05), **HC**: healthy control, **DC**: diabetic control, **D+MICT**: diabetes + moderate-intensity continuous training, **D+HIIT**: Diabetes+ High-intensity interval training, **FBG**: Fasting blood glucose, **HOMA-IR**: Homeostatic Model Assessment of Insulin Resistance, TTE: Time to exhaustion, (*n* = 8).

Regarding the body weight of rats, one-way ANOVA showed a significant difference between groups (F = 130.8, *p* = 0.001, ES = 0.93). Post-hoc analysis showed no significant difference between HC and DC groups. However, the D+MICT and D+HIIT groups had significantly lower values than the DC group, with decreases of 14.19%, MD: 54.38, 95% Cl [37.87, 70.88], *p* = 0.001 and 26.14%, MD: 98.88, 95% Cl [82.37,115.4], *p* = 0.001, respectively. The results also showed that the average weight in the D+HIIT group was significantly lower than in the D+MICT group (*p* < 0.001, -13.08%), (Table 2).

One-way ANOVA analysis of FBG showed that there was a significant difference between groups (F = 262.3, *p* = 0.001, ES = 0.96). In the DC group, it was approximately 3.8-fold higher compared to HC (MD: -16.93, 95% CI [-15.29, -18.57], *p* < 0.001). While in the D+MICT and D+HIIT groups, it was lower by -28.70%, MD:6.12, 95% CI [4.48, 7.76], *p* = 0.001 vs. -39%, MD:9.07, 94% CI [7.43, 10.72], *p* = 0.001 compared to the DC group. FBG levels in the D+HIIT group also were significantly lower by -19.37% compared to the D+MICT group (MD: 2.95, 95% CI [1.31, 4.59], *p* = 0.001), (Table 2**).**

HOMA-IR index showed a significant difference between groups (F = 87.54, *p* = 0.001, ES = 0.90). The DC group was significantly higher than HC (10.06-fold, MD: -5.83, 95% CI [-4.83, -4.83], *p* = 0.001), while D+MICT and D+HIIT groups were lower than the DC group by -50.23%, MD: 3.22, 95% CI [2.21,4.22], *p* = 0.001 and − 68.17%, MD: 4.37, 95% CI [3.37, 5.37], *p* = 0.001. There was also a significant 36.05% lower difference with D+MICT compared to D+HIIT (MD: 1.15, 95% CI [0.15, 2.15], *p* = 0.018), (Table 2**).**

### The effect of exercise modality on fission proteins in VAT

**Fig. 2:**
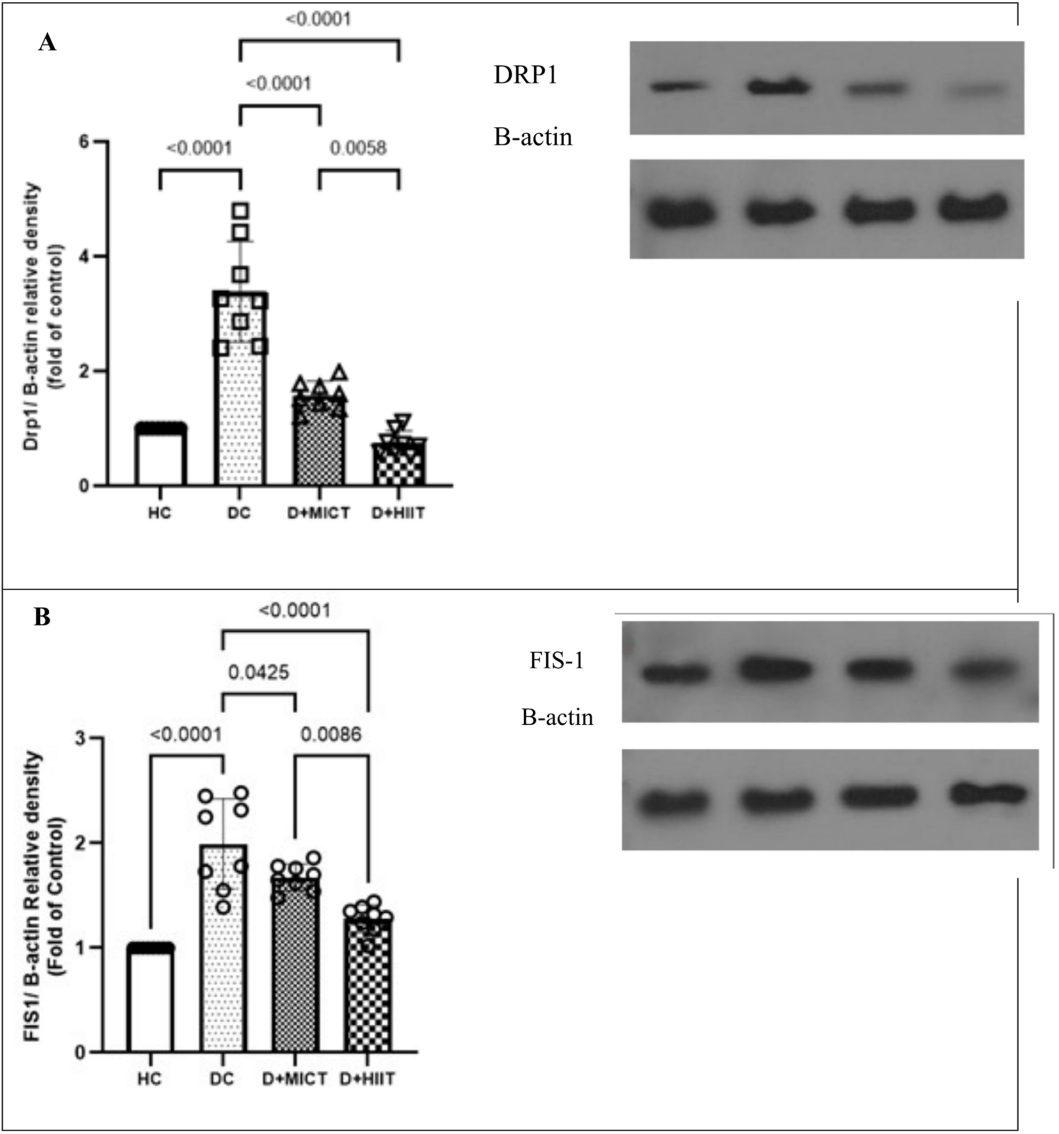
**A**, **B** The effect of moderate-intensity continuous training (MICT) and high-intensity interval training (HIIT) on the expression levels of mitochondrial fission proteins (DRP-1) and (FIS1) in visceral adipose tissue (VAT). **A** Representative Western blot bands of DRP-1 and FIS-1 proteins in four groups: healthy control (HC), diabetic control (DC), diabetes + MICT (D+MICT), and diabetes + HIIT (D+HIIT). **B** Quantitative analysis of DRP1 protein levels normalized to β-actin. **C** Quantitative analysis of FIS1 protein levels normalized to β-actin. Data are presented as mean ± SD (*n* = 8 per group). Statistical analysis was performed using one-way ANOVA followed by Tukey post hoc test. *P* < 0.001.

Based on ANOVA analysis, significant differences in DRP1 protein levels were observed between groups (F = 52.11, *p* = 0.001, ES = 0.84) (Fig. 1). Specifically, T2D led to a significant increase in DRP1 protein levels and consequently increased Fission activity in VAT of T2D rats. This increase was 2.38-fold in the diabetic group (DC) compared to the healthy control group (HC) MD: -2.38, 95%CI [1.00, 4.11], *p* = 0.001. However, in the groups treated with different exercise training, Drp1 levels and Fission activity were significantly reduced. In the D+MICT and D+HIIT group, a respective 53.55%, MD: 1.80, 95% CI [1.18, 2.43], *p* = 0.001 and 77.81%, MD: 2.63, 95% CI [2.01, 3.25], *p* = 0.001 decrease was observed compared to the DC group. Furthermore, a comparison between the two different types of exercise training showed that DRP1 protein levels in the D+HIIT group was 22.45% lower than that in the D+MICT group, MD: 0.82, 95%CL [0.58,1.78], *p* = 0.005), (Fig. 2, A**).**

Regarding FIS1 protein levels, ANOVA revealed a significant difference between groups (F = 27.93, *p* = 0.001, ES = 0.74). Post-hoc tests showed a 99% increase in FIS1 protein levels in DC vs. HC (MD: -0.99, 95% CI: [1:00, 2.35], *p* = 0.001). While the FIS1 levels in the D+MICT and D+HIIT were significantly lower than those in the DC group (16.08%, MD: 0.32, 95% CI [0.008, 0.632], *p* < 0.042 and 36.18%, MD: 0.71, 95% CI [0.40, 1.02], *p* = 0.001. The levels in D+HIIT were lower than the D+MICT (-23.95%, MD: 0.39, 95%CL (0.08,0.70), *p* = 0.008), (Fig. 2, B).

### Effect of exercise modality on VAT fusion protein levels

**Fig. 3:**
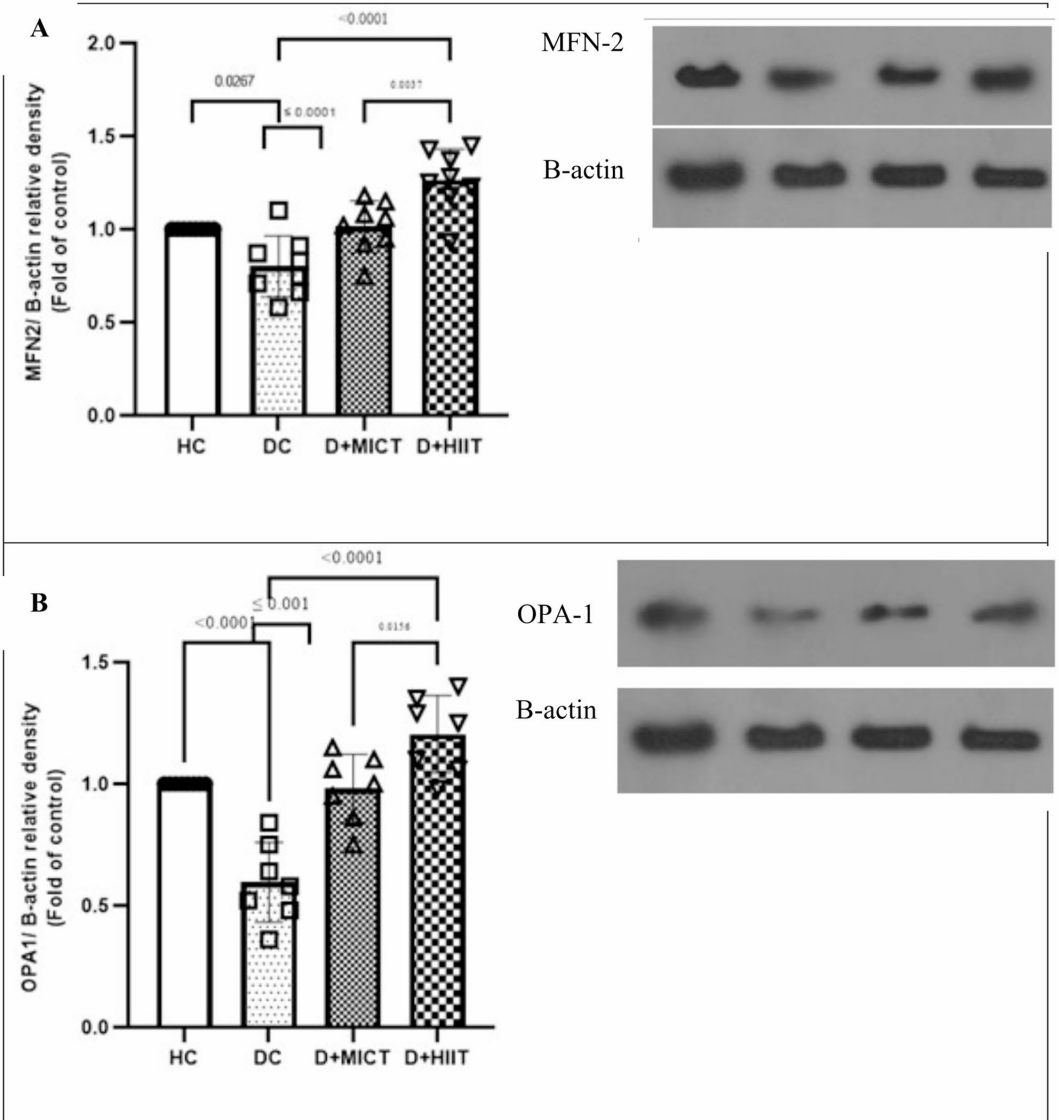
**A**, **B** The effect of moderate-intensity continuous training (MICT) and high-intensity interval training (HIIT) on the expression levels of MFN2 and OPA1 in visceral adipose tissue (VAT). **A** Representative Western blot bands of MFN-2 and OPA-1 proteins in four groups: healthy control (HC), diabetic control (DC), diabetes + MICT (D+MICT), and diabetes + HIIT (D+HIIT). **B** Quantitative analysis of MFN-2 protein levels normalized to β-actin. **C** Quantitative analysis of OPA-1 protein levels normalized to β-actin. Data are presented as mean ± SD (*n* = 8 per group). Statistical analysis was performed using one-way ANOVA followed by Tukey post hoc test.

ANOVA showed a significant difference in the protein levels of MFN2 across groups (F = 15.70, *p* = 0.001, ES = 0.62), with T2DM significantly reducing VAT fusion in the DC group compared to HC through a decrease in the protein content of MFN2 (-20%, MD: 0.19, 95% CI [0.01, 0.37], *p* < 0.02) (Fig. 2). In both the D+MICT and D+HIIT groups, levels were significantly higher than in the DC group (by 26.25%, MD: -0.21, 95% CI [-0.03, -0.39], *p* = 0.02 and 57.50%, MD: -0.46, 95% CI [-0.28, -0.64], *p* = 0.001. Additionally, the D+HIIT group showed significantly higher levels than the D+MICT group (24.75%, MD: -0.25, 95% CI [-0.07, -0.43], *p* < 0.003), (Fig. 3A). Furthermore, OPA1 protein levels was reduced by 41% in the DC group compared to the HC group (MD: 0.40, 95% CI [0.22, 0.58], *p* < 0.001). However, it was significantly higher in the D+MICT and D+HIIT groups compared to the DC group, with increases of 66.10%, MD: -0.38, 95% CI [-0.19, -0.57], *p* = 0.001 and 103.39%, MD: -0.60, 95% CI [-0.42, -0.79]. Additionally, the D+HIIT group showed significantly higher OPA1 levels than the D+MICT group (22.45%, MD: -0.22, 95% CI [-0.03, -0.41], *p* < 0.015), (Fig. 3, B).

### The effect of two types of MICT and HIIT on TTE of type 2 diabetic rats

**Fig. 4:**
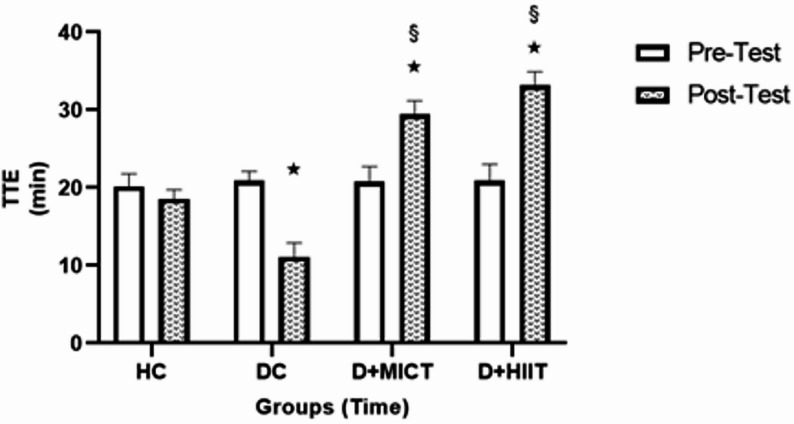
The effect of moderate-intensity continuous training (MICT) and high-intensity interval training (HIIT) on time to exhaustion (TTE) in type 2 diabetic rats. Bar graph represents TTE values (in seconds) measured after the 8-week intervention in four groups: healthy control (HC), diabetic control (DC), diabetes + MICT (D+MICT), and diabetes + HIIT (D+HIIT). Data are presented as mean ± SD (*n* = 8 per group). Statistical analysis was performed using one-way ANOVA followed by Tukey post hoc test. (§): Significantly vs. group DC, (*): Significantly vs. group HC. *P* < 0.001.

TTE was 40.10% lower in the DC group compared to the HC group (-39.02%, MD = -7.44 min, 95% CI [-5.34, 9.55], *p* = 0.001), while TTE was significantly higher in the D+MICT and D+HIIT groups compared to the DC group (by 164.33%, MD: -18.29, 95% CI [-16.19, -20.40] and 198.20%, MD: -22.06, 95% CI [-19.95, -24.16], with no significant difference between the two exercise groups (Fig. 4).

## Discussion

The present study aimed to compare the effects of two different types of exercise training on the content of mitochondrial dynamic proteins in VAT of Wistar Rats with type 2 diabetes. The results showed that type 2 diabetes increased the content of mitochondrial fission-related proteins including FIS1 and DRP1 (99% and 238%, respectively) and decreased the content of fusion-related proteins including MFN2 and OPA1 (20% and 10.40%, respectively). Interestingly, both types of exercise training (continuous moderate-intensity and intermittent high-intensity) were able to significantly improve these diabetic-induced disorders. Specifically, both training methods significantly reduced the levels of fission proteins (FIS1 and DRP1) and increased the content of fusion proteins (MFN1 and OPA1), such that the levels of these proteins returned to values ​​close to those of the healthy control group. These findings indicate that exercise interventions can be considered as effective strategies to modulate mitochondrial dynamic disorders caused by type 2 diabetes.

T2DM can increase mitochondrial fission and damage in several ways, including oxidative stress, inflammation, and insulin resistance. Increased blood sugar levels damage mitochondrial DNA and proteins through high levels of reactive oxygen species (ROS), which leads to mitochondrial dysfunction and an increase in proteins involved in mitochondrial fission (DRP-1 and FIS-1) [27]. In vivo studies in skeletal muscle of diet-induced obese (ob/ob, C57BL/6) mice show an imbalance in mitochondrial fusion and fission, where DRP1-dependent fission regulates the insulin pathway and is associated with insulin resistance [28]. T2DM has been associated with mitochondrial network fragmentation in the myocardium, accompanied by a marked decrease in *MFN1* expression [29]. Similarly, studies have shown that OPA1 depletion in mouse pancreatic β cells impairs insulin secretion, reduces glucose-stimulated ATP production, and induces hyperglycemia, highlighting the broader role of mitochondrial dynamics in T2DM pathophysiology. These changes were associated with defects in the activity of complex IV of the electron transport chain, while insulin enhances mitochondrial fusion and the NFκB signaling pathway in skeletal muscle cells and cardiomyocytes by increasing Opa1 and regulating mTOR [30]. This imbalance between fission and fusion caused by T2DM leads to mitochondrial fragmentation in adipose tissue, which leads to functional impairments including decreased ATP production, increased ROS production, insulin resistance, and abnormal lipid accumulation and inflammation, which further contribute to the systemic complications of T2D.

Our data on MICT and HIIT training interventions caused a decrease in DRP1 protein (53.55%, 77.81%, respectively) and a decrease in FIS1 (16.08%, 36.18%, respectively) compared to the DC group. This was consistent with the results of Li et al. [20], who showed 12 weeks of MICT and HIIT training reduced cognitive function decline in the hippocampus in APP/PS1 transgenic mice, which may be related to the improvement of mitochondrial morphology and dynamics. Studies on the effects of both MICT and HIIT training on mitochondrial dynamic indices are limited, with most studies focused on the effects of HIIT training on proteins involved in mitochondrial dynamics. However, Taklimi et al. found both HIIT and MICT training increased DRP1 factors in skeletal muscle tissue of rats, and this increase was greater in HIIT training than in MICT [18]. The finding that HIIT and MICT training increased DRP1 expression in muscle tissue in mice (Taklimi et al., 2021), while in the present study a decrease in this factor was observed in VAT, may be due to intrinsic functional and metabolic differences between these two tissues. Studies have shown that adipose tissue and skeletal muscle show contrasting responses to metabolic stimuli (Smith et al., 2020). In muscle tissue, the increase in DRP1 is likely to reflect a process of mitochondrial remodeling to meet oxidative demands [31], while a similar decrease in VAT may indicate improved metabolic function and reduced oxidative stress [32, 33]. These tissue-dependent differences are consistent with the findings of Jornayvaz et al. (2010) who showed that mitochondrial regulation in different tissues follows distinct mechanisms [34].

Banali and et al. (2024) [35], Tincknell and et al. (2023) [36], Choobineh and et al. (2024) [37], in their studies, consistent with the results of the present study, showed that short-term periods (8 weeks) increased FIS1 and DRP1 indices in animal samples. Several studies are consistent with the findings of the present study that FIS1 and DRP1 indices are increased after short-term (8 weeks) periods of MICT or HIIT. Specifically, Bendley et al. (2024) showed in a study on Wistar rats that moderate-intensity interval training increased the expression of these mitochondrial fission markers in the soleus muscle. Also, Tincknell et al. (2023) observed an increase in mitochondrial regulatory proteins, including fission factors, in skeletal muscle tissue after high-intensity interval training in a study on diet-induced obesity in C57BL/6 mice. In addition, Choubine et al. (2024) reported protective changes in mitochondrial dynamic proteins in cardiac tissue after high-intensity interval training in rats. Interestingly, another study also reported an increase in DRP1 content after a 10-week period of MICT and HIIT training [18]. These consistent findings across different animal models (rats and mice), different time periods (8–10 weeks), and different tissues (skeletal and cardiac muscle) support the results of the present study on the effect of training on the mitochondrial fission apparatus. Also, in the study of Pengam et al.., an increase in *FIS1* mRNA and *DRP1* levels in the long toe muscle of Wistar rats was observed after 6 weeks of MICT and HIIT training, and this increase was greater in the HIIT training group [17], which was inconsistent with the results of the present study. This difference can be explained by different samples or different training periods. In the studies of Pengam et al.., the rats were healthy and without any disease, while we used T2DM induced rats. In this regard, we can point out the possible factors involved in the results of the present study, including the T2DM induction model [17]. Our combined HFT + STZ model probably caused insulin resistance via a high-fat diet and beta cell damage by STZ respectively [38], leading to significant changes in mitochondrial pathways and increased expression of mitochondrial dynamics proteins such as FIS1 and DRP1 in adipose tissue, potentially driven by heightened oxidative stress and systemic inflammation [39].

Our observed reduction in FIS1 and DRP1 induced by both HIIT and MICT exercise interventions may be due to changes in biological pathways related to mitochondrial regulation, such as PGC-1α and AMP-activated protein kinase (AMPK) activation, which play an important role in the balance of mitochondrial dynamics. Exercise has been reported to activate AMPK, which can regulate mitochondrial dynamics by inhibiting Drp1 activity and potentially promoting fusion through MFN-2 [40]. PGC-1α regulates mitochondrial biogenesis and function, and both HIIT and MICT can enhance this process by upregulating PGC-1α, thereby reducing oxidative stress and improving energy balance [41]. This may have led to the reduction of FIS-1 and Drp-1 expression in VAT [42]. Supporting this, eight weeks of HIIT training increased PGC-1a in the gastrocnemius muscle of T2DM mice, thereby improving mitochondrial dynamics [10]. However, it can be said that the present findings in the type 2 diabetic rat model indicate that exercise may affect the expression of mitochondrial dynamic proteins through potential pathways such as PGC-1α and AMPK, although these mechanisms were not directly measured in our study.

We also showed that both MICT and HIIT increased mitochondrial fusion-related indices, including MFN-2 (by 26.25% and 57.50%) and OPA1 (by 66.10% and 103.39%), compared to the DC group, with greater increases observed in the HIIT group than in the MICT group for both MFN-2 (24.75%) and OPA1 (22.45%). These results were consistent with the results previous studies [15, 18, 20, 43–45]. Except for Pengam et al. and Taklimi et al. [17, 18], other studies have investigated the effect of only HIIT training on mitochondrial dynamic factors. In Pengam and Taklimi’s studies, both HIIT and MICT training increased MFN-2 and OPA-1 in obese rats, with the effects of HIIT being greater (24.75% vs. 22.45%; respectively). They also showed that the content of mitochondrial biogenesis regulatory proteins, PGC-1a and AMPK, increased simultaneously with mitochondrial dynamic factors. This activation probably leads to an increase in the expression of mitochondrial fusion-related genes and a decrease in the expression of fission-related genes. The findings of the present study indicate that this activation resulted in significant changes in the expression of genes regulating mitochondrial dynamics. Specifically, we observed that the expression of genes related to mitochondrial fusion, including MFN2 and OPA1, was significantly increased. In contrast, the expression levels of genes involved in mitochondrial fission, such as DRP1 and FIS, showed a significant decrease. This pattern of gene expression changes, which is consistent with previous studies such as Ding and et al. (2010), could indicate a tendency for cells to enhance fusion processes and weaken fission pathways in response to exercise intervention [46]. These changes likely reflect metabolic adaptations and improved mitochondrial function as a result of training.

Another possible mechanism is the effects of both HIIT and MICT training on the regulation of inflammatory pathways, as regular exercise can reduce ROS production and suppress inflammatory pathways via NF-κB, reducing mitochondrial fission and improving overall mitochondrial function [47].

Furthermore, the improvement of mitochondrial dynamic balance due to exercise training can be related to the improvement of insulin sensitivity. In this regard, we showed that both HIIT and MICT significantly reduced glucose levels (by 39% and 28.70%, respectively) and the HOMA-IR index (by 68.18% and 77.27%) compared to the DC group. These reductions in glucose and insulin levels suggest that both training modalities alleviate metabolic stress on mitochondria, contributing to improved mitochondrial quality. This effect may be due to an increase in glucose consumption by skeletal muscles, improved fat oxidation, and reduced lipotoxicity [48].

Our improved performance and enhanced metabolic efficiency findings could be explained by the increased TTE in the D+MICT and D+HIIT groups, as the results showed a 164.33% MICT and 198.20% HIIT increase in TTE compared to the DC group. This was consistent with previous studies showing the enhancement of TTE induced by MICT [23] and HIIT [49, 50] in T2DM rats. However, the increased TTE in the HIIT and MICT training groups indicated improved endurance and metabolic efficiency, which could be due to increased mitochondrial number and quality, improved oxidative capacity, and better regulation of energy production [51].

Although we did not measure PGC-1α and AMPK, given their role in regulating mitochondrial dynamics, it can be assumed that these pathways played a role in the decrease in FIS-1 and Drp-1 and the increase in MFN-2 and OPA-1 induced by MICT and HIIT training. These results emphasize the importance of investigating the biological mechanisms associated with exercise training in future research.

The present study has strengths such as a comparative design between two types of exercise training (MICT and HIIT) in an animal model of type 2 diabetes, the use of precise molecular markers related to mitochondrial dynamics (DRP-1, FIS-1, MFN-2, OPA-1) in VT. Also, the investigation of the effects of exercise on metabolic variables (blood glucose, HOMA-IR, TTE) are other strengths of this study. A major limitation of this study is the reliance solely on measuring the total levels of DRP1, FIS1, MFN2, and OPA1 proteins by Western blot. This method, although providing valuable information on molecular changes, cannot directly assess the functional status of mitochondrial dynamics (such as DRP1 phosphorylation, mitochondrial network morphology, subcellular distribution, or mitophagy activity); therefore, the observed changes at the protein level do not necessarily imply functional restoration or improvement of mitochondrial dynamics and should be interpreted with caution. In addition, the lack of direct measurement of key mediator proteins (such as PGC-1α and AMPK), the exclusive use of an animal model, and the absence of a combined exercise group or different exercise intensity/duration groups limit the generalizability of the results to humans. However, this study provides valuable evidence of the positive effect of exercise on the levels of mitochondrial fission and fusion regulatory proteins in a type 2 diabetes model and provides a good basis for future research, especially in human models.

## Conclusion

We showed that T2DM causes an imbalance in Fission and Fusion in the VAT of rats. Both HIIT and MICT training can significantly improve mitochondrial function and metabolic status in people with type 2 diabetes by regulating the levels of proteins that regulate mitochondrial fission and fusion and improving insulin sensitivity. Also, it seems that the regulation of the balance of mitochondrial fission and fusion in the VAT of T2DM rats was better in HIIT training than in MICT training. Overall, these results indicate the importance of exercise training in controlling T2DM and improving its associated complications.

## Supplementary Information

Below is the link to the electronic supplementary material.

Supplementary Material 1

## Data Availability

The data supporting this study’s findings are available from the corresponding author upon reasonable request.

## Abbreviations

T2DM: Type 2 diabetes
VAT: Visceral adipose tissue
HFD: High-fat diet
STZ: Streptozotocin
AT: Adipose tissue
FIS1: Fission-1
Drp1: Dynamin-related protein-1
Opa1: Optic atrophy 1
Mfn2: Mitofusin-2
MICT: Moderate-intensity continuous training
HIIT: High-intensity interval training
TTE: Time to exhaustion
V_max_: Maximum running speed
FBG: Fasting blood glucose
