# Supplementary material for: Effects of MICT and HIIT on mitochondrial dynamics-related proteins in visceral adipose tissue of type 2 diabetic rats
Source: Biol Res. 2026 Apr 24;59:37. doi: 10.1186/s40659-026-00694-x (PMC13255375; doi:10.1186/s40659-026-00694-x)
Supplement: Supplementary file 1 — Supplementary Material 1 [file 40659_2026_694_MOESM1_ESM.docx]

**Supplementary Figure S1: Color Western blot for DRP1**

| DRP1 | 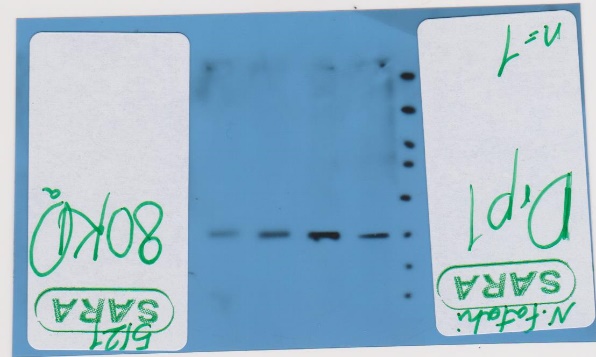 | 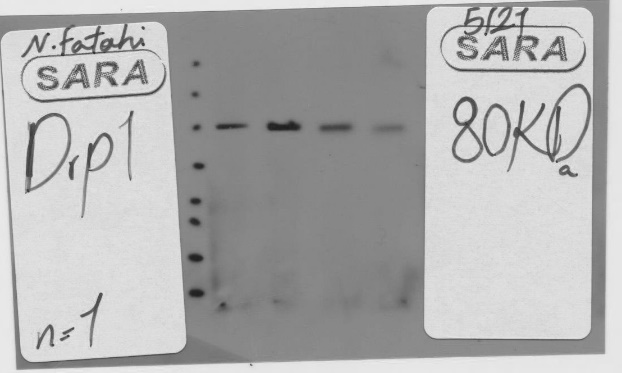 |
| --- | --- | --- |
| B-actin | 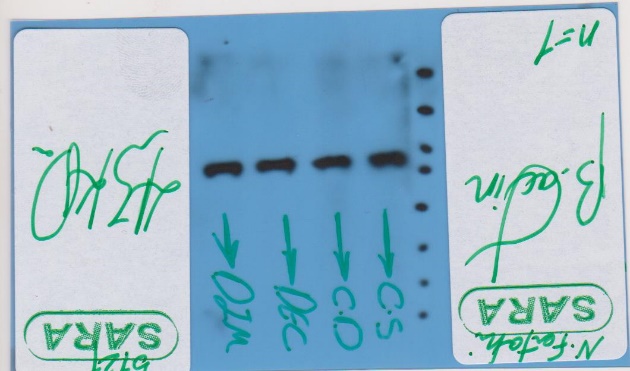 | 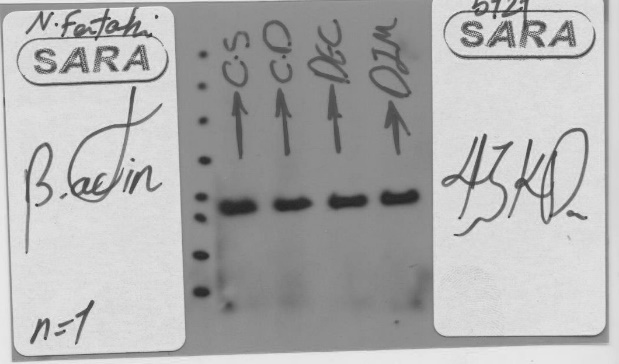 |

**Supplementary Figure S2: Color Western blot for FIS1**

| FIS1 | 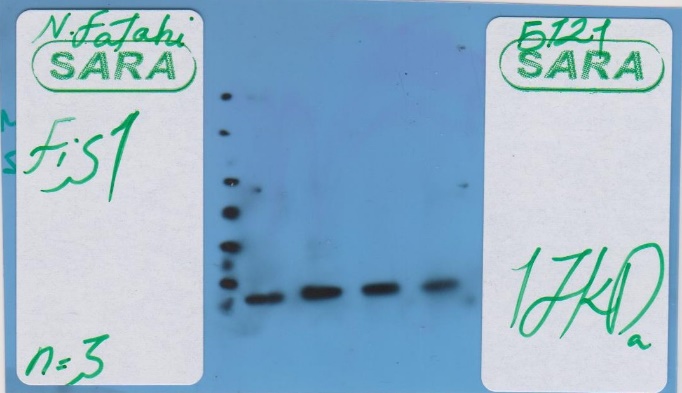 | 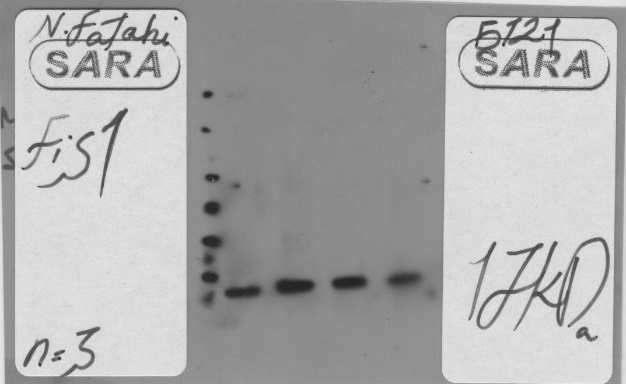 |
| --- | --- | --- |
| B-actin | 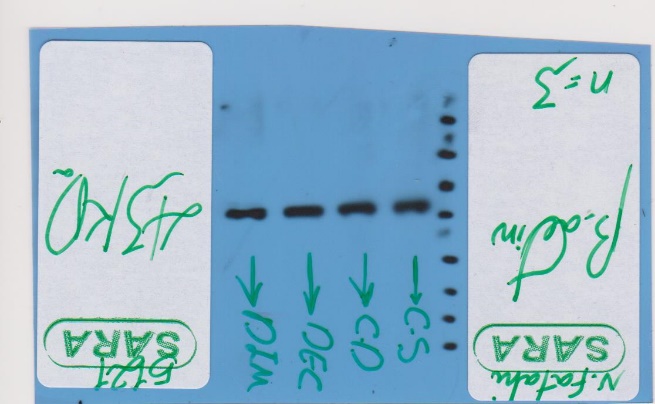 | 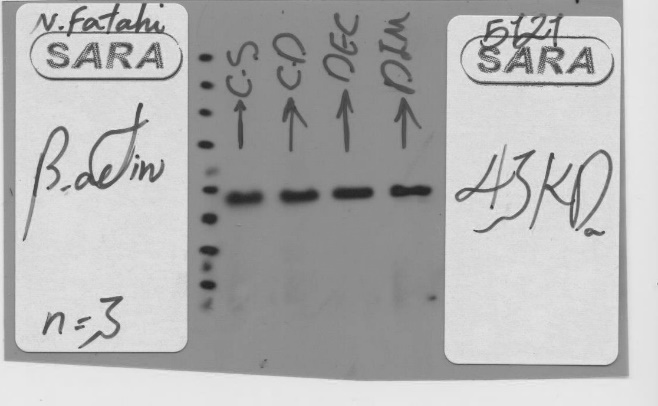 |

**Supplementary Figure S3: Color Western blot for MFN2**

| MFN2 | 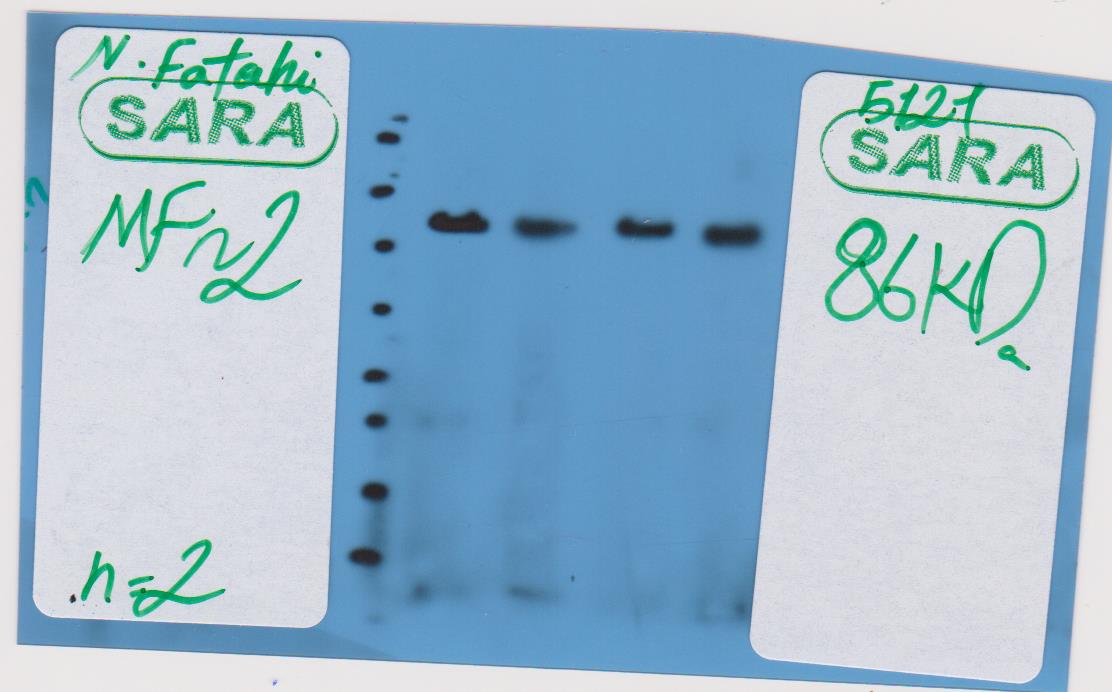 | 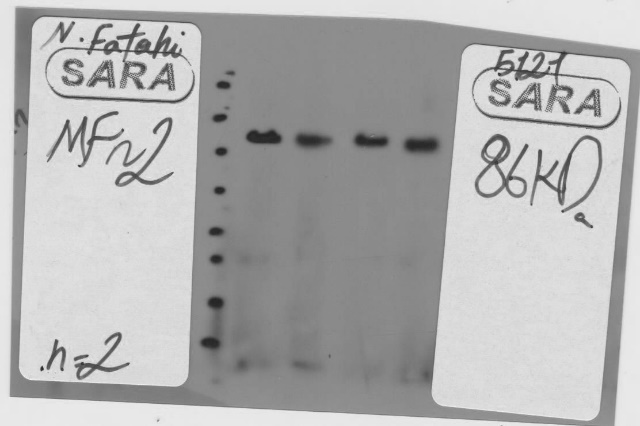 |
| --- | --- | --- |
| B-actin | 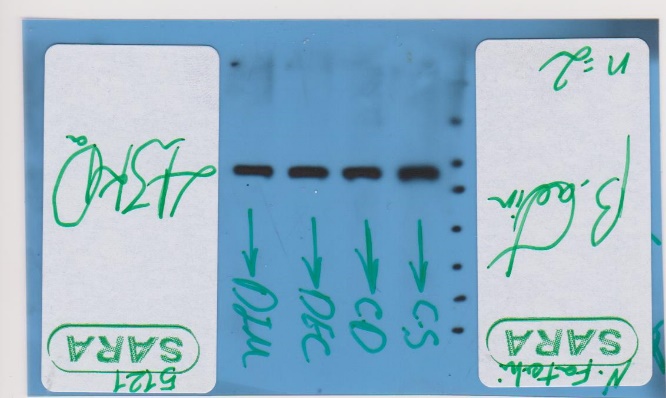 | 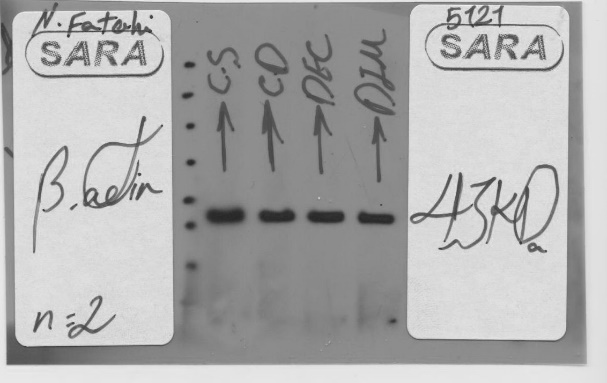 |

**Supplementary Figure S4: Color Western blot for OPA1**

| OPA1 | 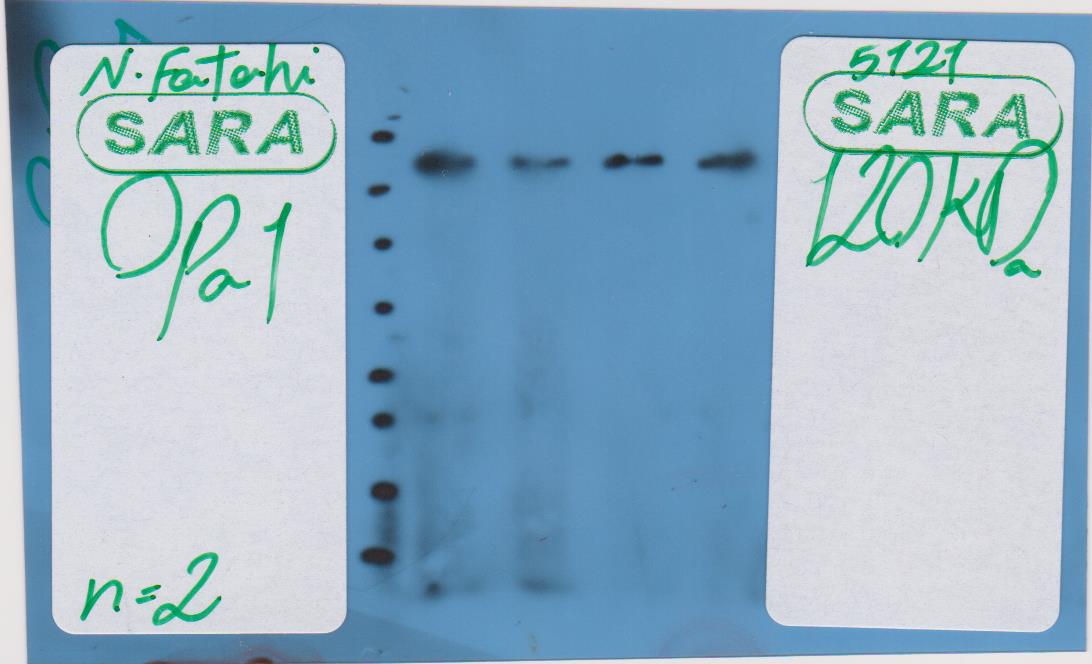 | 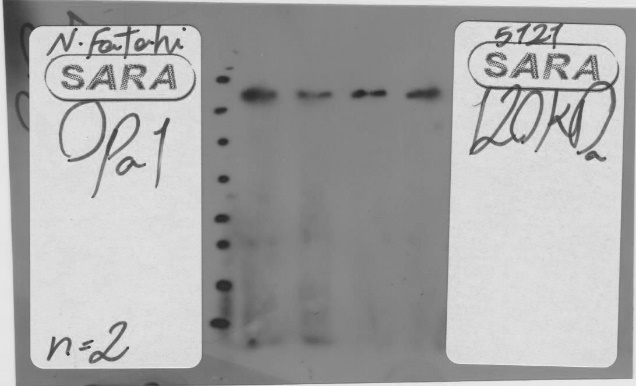 |
| --- | --- | --- |
| B-actin | 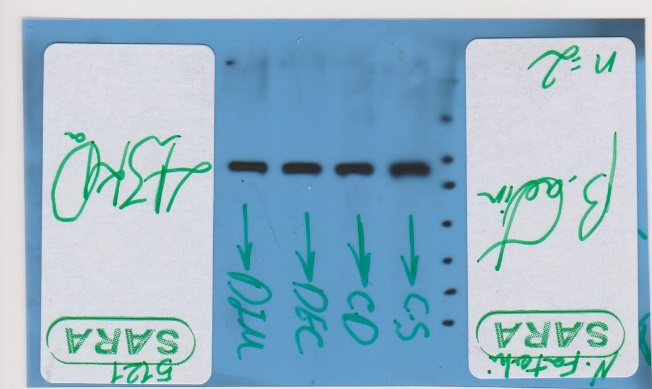 | 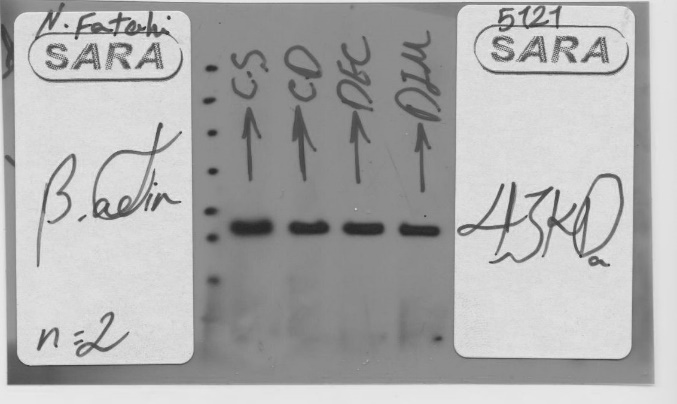 |
